# Evolution of Three Parent Genes and Their Retrogene Copies in *Drosophila* Species

**DOI:** 10.1155/2013/693085

**Published:** 2013-06-05

**Authors:** Ryan S. O'Neill, Denise V. Clark

**Affiliations:** Department of Biology, University of New Brunswick, 10 Bailey Drive, Fredericton, NB, Canada E3B 5A3

## Abstract

Retrogenes form a class of gene duplicate lacking the regulatory sequences found outside of the mRNA-coding regions of the parent gene. It is not clear how a retrogene's lack of parental regulatory sequences affects the evolution of the gene pair. To explore the evolution of parent genes and retrogenes, we investigated three such gene pairs in the family Drosophilidae; in *Drosophila melanogaster*, these gene pairs are *CG8331* and *CG4960*, *CG17734* and *CG11825*, and *Sep2* and *Sep5*. We investigated the embryonic expression patterns of these gene pairs across multiple *Drosophila* species. Expression patterns of the parent genes and their single copy orthologs are relatively conserved across species, whether or not a species has a retrogene copy, although there is some variation in *CG8331* and *CG17734*. In contrast, expression patterns of the retrogene orthologs have diversified. We used the genome sequences of 20 *Drosophila* species to investigate coding sequence evolution. The coding sequences of the three gene pairs appear to be evolving predominantly under negative selection; however, the parent genes and retrogenes show some distinct differences in amino acid sequence. Therefore, in general, retrogene expression patterns and coding sequences are distinct compared to their parents and, in some cases, retrogene expression patterns diversify.

## 1. Introduction

Gene duplication plays a major role in evolution by expanding  gene families and facilitating the diversification of  gene function. Following  duplication, gene copies can diverge in function. Retroduplication occurs when mRNA from a parent gene is reverse-transcribed and inserted into the genome, producing a new retrogene copy that lacks the regulatory elements and introns of the parent [1, 2]. The lack of parental regulatory elements in a new retrogene is often associated with a lack of function and pseudogenization; however, those retrogenes that are transcribed presumably lack the expression pattern of their parents and may therefore acquire novel functions [3].

Genome-wide studies have provided insights into the evolutionary outcome of this initial asymmetry between parent gene and retrogene regulatory elements and expression patterns, including several studies focusing on protein-coding parent genes and retrogenes in *Drosophila*. Expression data from *Drosophila melanogaster* show that retrogenes tend to be expressed at a lower level and in fewer tissues than their parents, with the exception that retrogenes tend to be more represented in testes than parent genes [4, 5]. The mean expression levels of retrogenes are not significantly different from all genes in *D. melanogaster* [5], suggesting that the loss of parental regulatory elements does not limit the expression of functional retrogenes. An investigation of retrogene regulatory elements in *D. melanogaster* by Bai et al. [6] indicated that retrogenes do not typically acquire regulatory elements from their parent genes. Cis-regulatory element prediction has been applied to retrogenes in *D. melanogaster*, but many of these putative elements are not conserved in other *Drosophila* species [7]. It is unclear whether or not the expression patterns of a parent gene and retrogene are conserved across species. Conservation of a retrogene's expression pattern would indicate an early establishment of function, whereas a lack of conservation would suggest diversification of function. Also, if a parent gene's expression pattern is conserved across species, regardless of whether or not the retrogene is present, then this would suggest that the retrogene has evolved a novel function rather than taking over part of the parent gene's function via subfunctionalization [8]. 

To gain insight into the evolution of retrogene expression, we examined three gene pairs that were previously identified in *D. melanogaster*, each consisting of a parent gene and retrogene [4, 5]. These *D. melanogaster* parent genes are *CG8331*, *CG17734*, and *Sep2*. The retrogene copies of these *D. melanogaster* parent genes are *CG4960*, *CG11825*, and *Sep5*, respectively. These gene names will be used herein for the orthologous genes in other *Drosophila* species. In each case, one copy is found on chromosome 2 and the other is found on chromosome 3 of *D. melanogaster*, which will facilitate genetic crosses and strain construction for future work. We chose autosomal gene pairs, rather than autosomal retrogenes with X-linked parents, in order to study diversification of gene duplicates more generally. It is thought that some of these latter duplicates were retained because they evolved male germline-specific functions that were not possible in the parent gene due to meiotic sex chromosome inactivation during spermatogenesis [9–12]. 

*CG8331* and *CG4960* are homologs of REEP5/6 (receptor accessory protein) in non-Arthropod Metazoa [13] and of Yop1p in *Saccharomyces cerevisiae*, an accessory protein to the Yip1 p Rab GTPase [14]. *CG17734* and *CG11825* encode hypoxia-induced gene domain (HIGD) homologs, originally identified as an upregulated gene during hypoxia [15] and later as a mitochondrial inner membrane protein involved with cell survival under stress [16]. *Sep2* and *Sep5* are members of the septin family of proteins [17]. Septin function is associated with plasma membrane and cortical cytoskeleton [18]. In *D. melanogaster*, septins are involved in many processes, including cytokinesis [19].

We searched the genomes of sequenced *Drosophila* species for the presence or absence of these three gene pairs. These results allowed us to infer where in the phylogeny the retroduplication occurred. Assuming the three retroduplications occurred on the most recent branch of the *Drosophila* phylogeny leading to retrogene-containing descendants, then the three retrogenes arose between 12.8–35.6 (*CG4960*), 35.6–41.3 (*CG11825*), and 62.2–62.9 (*Sep5*) million years ago [20]. Thus, for each case, there are *Drosophila* species with sequenced genomes that do not have the retroduplication [21]. Comparisons between parent genes and their single-copy orthologs should reveal how the parent gene function has changed following retroduplication [22]. 

Here we show that the orthologs of these parent genes and retrogenes are transcribed in 10 *Drosophila* species, indicating that they have not become pseudogenes. Embryonic expression patterns of parent genes, retrogenes, and singletons were determined by *in situ* hybridization. We find that the embryonic expression patterns of the parent gene *Sep2 *are conserved across the species investigated, whereas *CG17734* and *CG8331* have some variation among species that do not correlate with retrogene presence or absence. The three retrogenes show evidence of expression pattern diversification among species. The coding sequences of the three gene pairs appear to be evolving predominantly under negative selection, with very little evidence of positive selection; however, the parent genes and retrogenes do show distinct differences in amino acid sequence that suggest functional diversification worthy of further exploration.

## 2. Materials and Methods

### 2.1. *Drosophila* Strains

The *Drosophila* species strains used to generate the 12 *Drosophila* genomes [21, 26, 27] were used in this study. *D. melanogaster* strain *y*;  *Gr*22*b* *Gr*22*d* *cn* *CG*33964^*R*4.2^ *bw* *sp*; LysC MstProx GstD5 Rh6 was obtained from Bloomington *Drosophila* Stock Center at Indiana University. *D. pseudoobscura* 14011-0121.94, *D. ananassae* 14024-0371.13, *D. erecta* 14021-0224.01, *D. mojavensis* 15081-1352.22, *D. sechellia* C 14021-0248.25, *D. simulans* w501 14021-0251.195, *D. virilis* 15010-1051.87, *D. willistoni* 14030-0811.24,and* D. yakuba* 14021-0261.01 were obtained from the *Drosophila* Species Center at UC San Diego, CA, USA.

### 2.2. Sequences

Coding and amino acid sequences of all transcripts for *CG8331*, *CG4960*, *CG17734*, *CG11825*, *Sep2*, and *Sep5* in *D. melanogaster* were obtained from FlyBase release FB2012_02 [28, 29]. To identify the parent gene transcript that likely gave rise to the retrogene, all pairwise alignments of coding sequences derived from alternative transcripts in *D. melanogaster* were generated using the Needleman-Wunsch algorithm [30]. The coding sequences of these homologous transcripts were used for further sequence analyses. 

Amino acid and coding sequences of the orthologs in other sequenced *Drosophila* species were obtained from FlyBase [28] using the coding sequences of the *D. melanogaster* genes as BLAST [31] queries. If a BLAST search resulted in a predicted gene model, then coding and protein sequences were obtained from that gene model; if no gene model existed for a particular search result, then coding and protein sequences were predicted using GeneWise [32]. If no ortholog was found in a particular species, we determined whether its absence was due to a deletion or an absence of genomic sequence data by performing BLAST searches, using the *D. melanogaster* genomic sequences flanking these genes as queries. In some cases, no BLAST hit corresponded to >100 kilo bases of sequence containing the gene in *D. melanogaster*, suggesting that the gene may be absent due to a gap in that species genome assembly. We chose to include sequences from all available sequenced *Drosophila* species to increase the power of our analyses for detecting selection acting on gene pairs. Accession numbers of the genes and genomes used are listed in Supplementary File 1 in Supplementary Material available online at http://dx.doi.org/10.1155/2013/693085.

Codon alignments of each gene pair were constructed by aligning protein sequences using Clustal Omega [33] with default settings, reverse-translating the protein alignment into a codon alignment with PAL2NAL [34], and then checking the alignments and removing codons that contained gaps or that were ambiguously aligned in some species [35]. For each codon alignment, MEGA5 [36] was used to determine the best model of sequence evolution and then construct a phylogenetic tree using maximum likelihood. Trees were visualized using iTOL [37, 38].

### 2.3. Quantitative Reverse Transcriptase PCR (qRT-PCR)

Total RNA was extracted from one pool of ten 2–4-day-old males or females using TRIzol (Invitrogen). RNA was DNase treated using RQ1 RNase-Free DNase (Promega). cDNA synthesis was performed using the reverse transcriptase (RT) enzyme mix included in the SuperScript III Platinum Two-Step qRT-PCR Kit (Invitrogen). Gene-specific primers were designed using Primer3 [39] for orthologous gene pairs and RpL32 in 10 *Drosophila* species (Supplemental File 1). Each qRT-PCR was performed with three technical replicates and one RT control using the Corbett Rotor-Gene 6000. Cross threshold (CT) values and PCR efficiencies were determined using LinReg, version 11.1 [40]. CT values of the technical replicates were averaged and efficiency corrected to 100% using the formula CT_100%efficiency_ = CT∗[log(1 + efficiency)/log(2)] [41]. Relative quantification (RQ) of genes was determined using the formula RQ = 2^(CTgeneA−CTgeneB)^ [41]. To compare transcripts within RNA samples, the ratios of parent gene to retrogene transcripts were calculated.

### 2.4. *In Situ* Hybridization

For each gene pair, we chose to investigate expression patterns in a subset of *Drosophila* species spread throughout the phylogeny. In each case, we included at least two species without the retrogene. DNA templates for sense and antisense RNA probe synthesis were amplified from cDNA using gene-specific primers (Supplemental File 1). A T7 RNA polymerase promoter sequence (TAATACGACTCACTATAG + A/G) was added to the 5′ end of either the forward primer to synthesize sense probes or the reverse primer to synthesize antisense probes. Templates were gel-purified, using QIAEX II Gel Extraction Kit (Qiagen), and sequenced using Sanger sequencing. Sense and antisense digoxigenin- (DIG-) labeled RNA probes were synthesized from purified probe templates using T7 RNA polymerase and DIG RNA labeling mix (Roche Applied Science). Probes were DNase-treated, precipitated overnight, and resuspended in DEPC-treated distilled water. Probe yield was estimated by spotting dilutions of probe, along with DIG-labeled control RNA (Boehringer Mannheim), onto positively charged nitrocellulose, incubating with Anti-DIG-AP antibody (Roche) and detecting using AP Buffer (100 mM NaCl, 50 mM MgCl_2_, 100 mM TrisCl pH 9.5, and 0.1% Tween-20) with NBT and BCIP (Roche).

Zero- to five-day-old flies were aged in bottles with food and live yeast paste for 3 days. Embryos were collected in Embryo Collection Cages (Genesee) on grape agar plates with live yeast paste for 9 or 18 hours (18 or 36 hours for *D. virilis*). Eggs were dechorionated with 50% bleach, then fixed in 4% formaldehyde in 1x PBS and heptane for 20 minutes, shaken in methanol, and stored at −20°C. *In situ* hybridization was performed according to Tautz and Pheifle [42], with modifications from [43, 44]. Embryos were warmed to room temperature and rehydrated by washing in 3 : 1 methanol : PBST, 1 : 3 methanol : PBST, and then PBST (1x PBS, 0.1% Tween-20). Embryos were fixed with 4% formaldehyde in PBST, digested for 4 minutes with 25 *µ*g/mL proteinase K (Merck), washed for 2 minutes with 2 mg/mL glycine in PBST, and fixed again with 4% formaldehyde in PBST. Embryos were washed with 50% hybridization solution (50% formamide, 5x SSC, 100 *µ*g/mL heparin, 100 *µ*g/mL sonicated salmon sperm DNA, and 0.1% Tween-20) in PBST for 5 minutes and then incubated for 2 hours at 56°C with hybridization solution that was boiled for 5 minutes and then chilled on ice for 5 minutes. Probe was added to hybridization solution at about 50 ng/mL, incubated at 80°C for 3 minutes, and chilled on ice for 5 minutes. Embryos were hybridized with this probe solution for 16 hours at 56°C, washed in progressive dilutions of hybridization solution and PBST, then washed with PBST. Embryos were cooled to room temperature and incubated with a 1 : 2000 dilution of anti-digoxigenin-AP antibody in PbT (1x PBS, 0.1% Triton X100, and 0.2% bovine serum albumin). Embryos were washed with PBST and then AP buffer, before staining with AP buffer containing NBT and BCIP. The color reaction was stopped by washing several times in PBST, then ethanol, then PBST, and the embryos were placed in a solution of 70% glycerol in PBS and stored at 4°C. Embryos were mounted on slides and viewed under differential interface contrast using the Leica DM RXA2 microscope. Individual embryos were staged according to Campos-Ortega and Hartenstein [24] and assessed for reproducible staining patterns. Photomicrographs were captured using a Leica DC500 camera and ThumbsPlus 4.0 software and were rotated and cropped using Fiji software. 

### 2.5. Analyses of Coding Sequences

Structural features of the proteins encoded by the genes in this study were predicted using TMHMM v. 2.0 [45] for transmembrane helices and Multicoil2 [46] for coiled coils. 

Nonsynonymous and synonymous substitution rates (dN and dS, resp.) and the ratio of dN/dS (*ω*) can be used to infer selection: *ω* < 1 implies purifying selection, *ω* = 1 implies neutral evolution, and *ω* > 1 implies positive selection. We used several methods to test for selection acting on the gene pairs in this study. Codon alignments, with ambiguities and gaps removed, were used in each test (Supplemental File 6). 

To test for positive selection acting specifically on either retrogenes or parent genes, we performed the branch-site test [47], implemented in the CODEML program of the PAML package version 4 [48]. The branch-site test considers variation in *ω* across branches and codons of a phylogenetic tree to determine whether the pattern of selection on a specified lineage (foreground) is significantly different from that on the rest of the tree (background). A maximum likelihood approach is used to compare different models and infer positive selection acting on the specified lineage. The null model allows only purifying or neutral selection. The alternative model constrains codons in the background to purifying or neutral selection, while allowing codons in the foreground to undergo purifying, neutral, or positive selection. If a likelihood ratio test (LRT) of the alternative versus the null model is significant, then positive selection on the foreground branches is inferred. Significance of the LRT is assessed using a chi-squared distribution with 1 degree of freedom. In cases where positive selection is inferred, the Bayes Empirical Bayes (BEB) method [49] is used to estimate which codons evolved under positive selection. For each PAML analysis, we submitted a custom tree that constrained the relationships between genes to the relationships between species on the *Drosophila* phylogeny [21, 23], thus assuming that each retrotransposition occurred on the most recent branch leading to the clade containing all species with the retrogene. We designated the branch giving rise to either the retrogene or the parent gene (excluding the singletons) as the foreground branch to test for positive selection on that clade.

We used the Datamonkey server [50, 51] to perform additional selection analyses. Substitution models were chosen for each gene pair using the model selection tool. Neighbour-joining trees generated on the Datamonkey server were used for the analyses. FEL [52] was used to detect sites evolving under negative selection in both the parent and retrogene. MEME [53] was used to detect sites that underwent episodic diversifying selection in a subset of lineages. We used a *P* value cutoff of 0.05 for both FEL and MEME analyses, which should be conservative based on simulations [52, 53].

Visual inspection of the multiple sequence alignments for each gene pair revealed some amino acid sites that were obviously different between the parent gene and retrogene, particularly in *Sep2* and *Sep5*, but which were not identified by the selection analyses listed above. Therefore, we manually identified sites with ≥80% amino acid conservation across parent genes (including singletons), ≥80% amino acid conservation across retrogenes, but which were different between the parent and retrogene. 

## 3. Results and Discussion

### 3.1. Determination of Paralogous Coding Sequences

**Figure 1:**
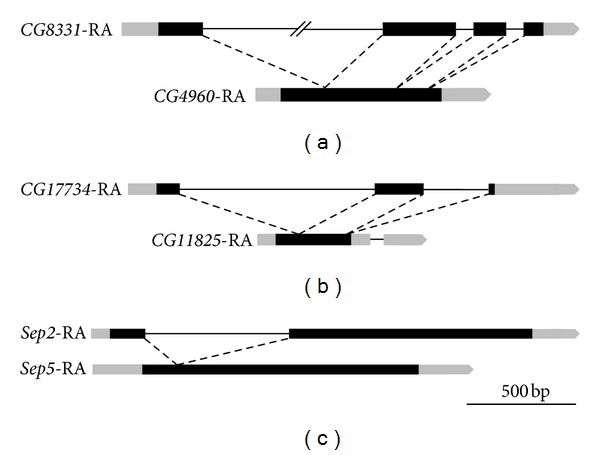
Three retroduplications in *D. melanogaster*. For each gene pair, coding sequences of transcripts were aligned to determine the most similar transcripts between parent gene and retrogene. Black bars indicate coding sequence, grey bars indicate untranslated regions, and thin lines indicate introns. Dashed lines show intron loss from parent gene to retrogene. Gene models were obtained from FlyBase. (a) *CG4960* is a retroduplication of either CG8331-RA (shown) or CG8331-RD, which encodes identical proteins. (b) CG17734-RA gave rise to *CG11825*. CG11825-RA is the retrogene transcript with the highest level of sequence identity to any transcript of *CG17734*. (c) *Sep2* encodes a single transcript. *Sep5* encodes 2 transcripts, Sep5-RA (shown) and Sep5-RB, which both contain identical coding sequence.

For each gene pair, alternative transcripts from the parent gene and retrogene in *D. melanogaster* were obtained from FlyBase [29], and pairwise sequence alignments of the coding regions from these transcripts were performed to identify the paralogous coding sequences. These paralogous coding sequences were used to determine the orthologous coding sequences of gene pairs in other *Drosophila* species. The three retroduplications are shown in Figure 1, and details of the coding sequence pairwise analyses are in Supplemental File 1. 

### 3.2. Identification of Orthologous Parent Genes and Retrogenes

**Figure 2:**
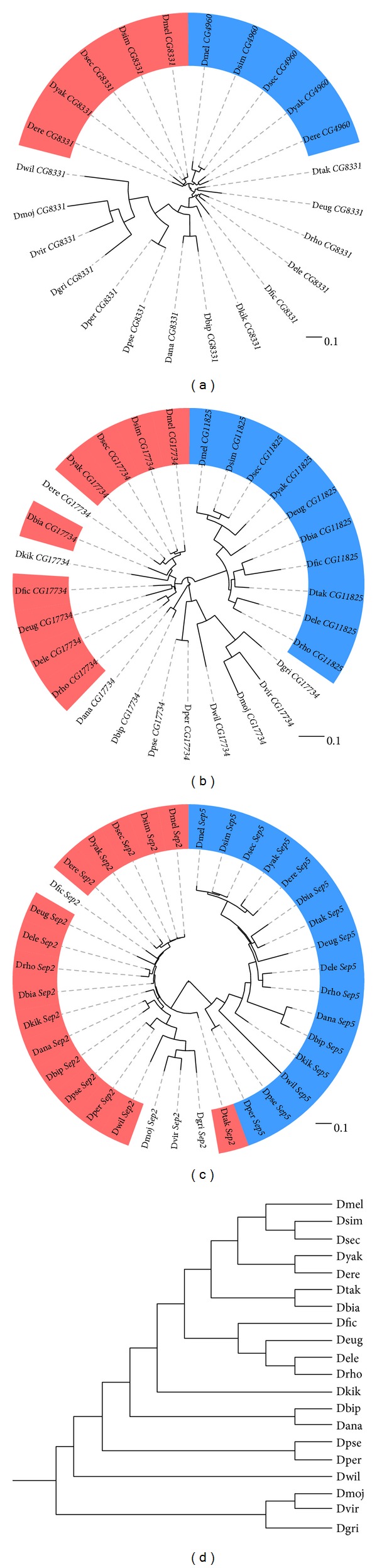
Phylogenetic analysis of three gene pairs shows that parent genes and retrogenes form independent clades. Maximum-likelihood trees were constructed from codon alignments of orthologs of each gene pair. Pink and blue highlightings correspond to parent genes and retrogenes, respectively. Trees are rooted to highlight relationships between parent genes, retrogenes, and singletons. (a) *CG8331* and *CG4960 *(*CG8331* is missing from Dbia due to a gap in the genome assembly); (b) *CG17734* and *CG11825* (*CG17734* is missing from Dtak due to a gap in the genome assembly, and *CG11825* was deleted in Dere); (c) *Sep2* and *Sep5* (*Sep5* is missing from Dfic due to a gap in the genome assembly); (d) cladogram showing phylogeny of sequenced *Drosophila* species, according to Yang et al. [23]. Dmel, *D. melanogaster*; Dsim, *D. simulans*; Dsec, *D. sechellia*; Dyak, *D. yakuba*; Dere, *D. erecta*; Deug, *D. eugracilis*; Dbia, *D. biarmipes*; Dtak, *D. takahashii*; Dfic, *D. ficusphila*; Dele, *D. elegans*; Drho, *D. rhopaloa*; Dkik, *D. kikkawai*; Dbip, *D. bipectinata*; Dana, *D. ananassae*; Dpse, *D. pseudoobscura*; Dper, *D. persimilis*; Dwil, *D. willistoni*; Dmoj, *D. mojavensis*; Dvir, *D. virilis*; Dgri, *D. grimshawi*.

We used BLAST [31] to identify orthologs of *CG8331*, *CG4960*, *CG17734*, *CG11825*, *Sep2*, and *Sep5* in the sequenced *Drosophila* species (Supplemental File 1). BLAST reported two copies of *CG11825* in *D. yakuba* with 100% nucleotide sequence identity, so only one copy was used for further analyses. BLAST also reported two copies of *CG17734* in *D. ficusphila*; one has a frameshift mutation near the 3′ end and so was excluded from further sequence analyses. *CG11825* appears to have been lost in *D. erecta*. Maximum-likelihood trees of each gene pair show that orthologous retrogenes form monophyletic clades, supporting a single retroduplication in each case (Figure 2). 

### 3.3. Investigation of Parent Gene and Retrogene Expression across Species

To assess functionality of *CG8331*, *CG4960*, *CG17734*, *CG11825*, *Sep2*, and *Sep5* in *Drosophila* species other than *D. melanogaster*, we confirmed their expression in adult males and females of 10 species using qRT-PCR (Supplemental Figure 5). In each species, the retrogenes showed lower expression compared to their parent genes. 

To explore the evolution of parent gene and retrogene expression patterns, embryonic expression patterns of the three gene pairs were detected by *in situ *hybridization of antisense RNA probes in various species. Sense-probe treatments were done in parallel to assess background staining (Supplemental File 7). Unless otherwise noted, background staining was not detected. We compared our embryonic expression patterns in *D. melanogaster* to those generated by the Berkeley Drosophila Genome Project (BDGP) [54, 55] when possible. Data from FlyAtlas [56] and the modENCODE developmental transcriptome of *D. melanogaster* [57] presented on FlyBase [29] provided additional information on gene expression (summarized in Supplemental File 1), complementing the qualitative expression patterns presented here.

### 3.4. Parent Gene *CG*8331 and Retrogene *CG*4960

**Figure 3:**
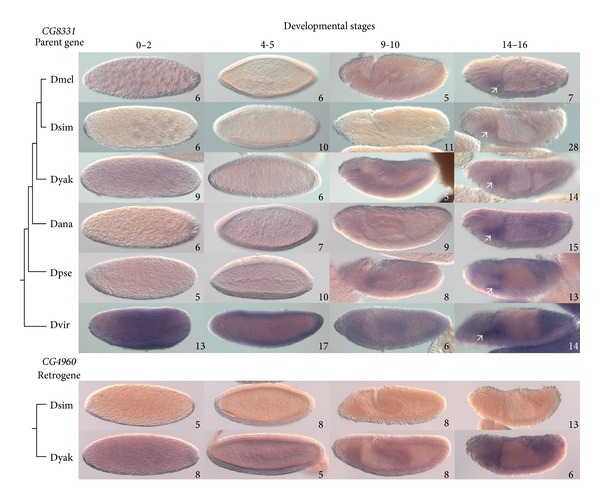
Expression patterns of *CG8331* and *CG4960*. Expression of *CG8331* and *CG4960* was detected in *Drosophila* species by *in situ* hybridization as shown in the top and bottom panels, respectively. Purple coloration indicates presence of mRNA. Cladograms show the relationships of the species investigated. Numbers on individual panels indicate the number of embryos assessed with consistent staining patterns. White arrows point to salivary glands. Stage ranges are according to [24]: 0–2, syncytial blastoderm; 3-4, cellular blastoderm; 9-10, during germ-band elongation; 14–16, final embryonic stages. Staining during stages 0–2 indicates presence of maternal transcripts [25]. Dmel, *D. melanogaster*; Dsim, *D. simulans*; Dyak, *D. yakuba*; Dana, *D. ananassae*; Dpse, *D. pseudoobscura*; Dvir, *D. virilis*.

*CG8331* transcript is detected in the salivary glands of late-stage embryos in all species examined (Figure 3). *D. melanogaster *also shows maternal transcript, which disappears by cellular blastoderm and low-level ubiquitous transcript appearing again at the end of gastrulation. *D. simulans* shows low-level ubiquitous transcript only detectable at the end of embryogenesis. *D. yakuba* also shows ubiquitous staining throughout embryonic development, although there was some background following the sense probe negative control. *D. ananassae*, *D. pseudoobscura*, and *D. virilis* also show ubiquitous staining throughout embryonic development. FlyAtlas found that *CG8331* is expressed in *D. melanogaster* larvae and adults in all tissues investigated, with the highest level of expression in salivary glands. The modENCODE developmental transcriptome of *D. melanogaster* shows that *CG8331* is highly maternally expressed, is reduced from cellular blastoderm through to gastrulation, and then peaks in expression during germ-band retraction, when the salivary glands develop [24]. These observations indicate that, except in *D. simulans*, *CG8331* has maternal expression and ubiquitous expression through early embryogenesis, and all species showed expression in salivary glands, with a lower level of ubiquitous expression in late embryogenesis.

*CG4960* was not investigated in *D. melanogaster* since no embryonic transcription was detected in the modENCODE developmental transcriptome. However, FlyAtlas and the modENCODE developmental transcriptome data indicate that *CG4960* has adult male testis-specific expression in *D. melanogaster*. Likewise, no embryonic expression was detected in *D. simulans* (Figure 3). However, *CG4960* is expressed ubiquitously throughout embryogenesis in *D. yakuba*, with dark staining in the midgut during later stages of embryonic development. These observations show that *CG4960* has diversified in expression pattern. 

Testis expression of *CG4960* appears conserved across species: Zhang et al. [58] found that *CG4960* is upregulated in *D. yakuba* males compared to females. Retrogenes are often expressed in testes in *Drosophila* [4, 5] and other species [59, 60]. The “out of the testis” hypothesis proposes that this is a common initial expression pattern for retrogenes, which could allow a retrogene to persist and evolve new expression patterns [59]. This was not found to be a trend for *Drosophila* retrogenes [4], although it could apply to individual cases of retroduplication. Bai et al. [6] showed that testes expression of *Drosophila* retrogenes can be due to genomic position within testis-biased gene neighborhoods. modENCODE tissue expression data presented on FlyBase shows that six out of eight of the neighboring genes within 20 kilobases flanking either side of *CG4960* are expressed higher in testis compared to other tissues, suggesting that *CG4960* is located within a testis-biased gene neighborhood. If the embryonic expression of *CG4960* in *D. yakuba* is a derived characteristic, it may be a good model for studying the “out of the testis” hypothesis.

#### 3.4.1. Parent Gene *CG*17734 and Retrogene *CG*11825

**Figure 4:**
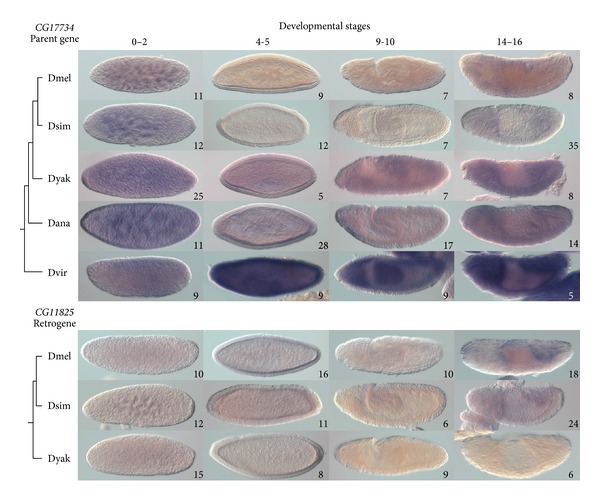
Expression patterns of *CG17734* and *CG11825. *Expression of *CG17734* and *CG11825* was detected in *Drosophila* species by *in situ* hybridization as shown in the top and bottom panels, respectively. Purple coloration indicates presence of mRNA. Cladograms show the relationships of the species investigated. Numbers on individual panels indicate the number of embryos assessed with consistent staining patterns. Stage ranges are according to [24]: 0–2, syncytial blastoderm; 3-4, cellular blastoderm; 9-10, during germ-band elongation; 14–16, final embryonic stages. Staining during stages 0–2 indicates presence of maternal transcripts [25]. Dmel, *D. melanogaster*; Dsim, *D. simulans*; Dyak, *D. yakuba*; Dana, *D. ananassae*; Dvir, *D. virilis*.

Maternal expression of *CG17734* was detected in all species investigated (Figure 4). In *D. melanogaster, CG17734* transcript is detected weakly during syncytial blastoderm and gastrulation, and the transcript is ubiquitous during late development. The modENCODE developmental transcriptome for *D. melanogaster* indicates that *CG17734* transcript is also present at relatively low to moderate levels from cellular blastoderm until late embryonic development. The BDGP found that, in *D. melanogaster*, *CG17734* is expressed maternally, is rapidly degraded during cellular blastoderm, and is expressed in the developing germline during gastrulation and later stages. In *D. simulans*, *CG17734* is also weakly expressed in the cellular blastoderm and throughout gastrulation and ubiquitously expressed during late embryogenesis. *CG17734* transcript is detected ubiquitously throughout embryogenesis in *D. yakuba*, *D. ananassae*, and *D. virilis*, but the intensity of the staining across embryonic stages in these species does not indicate a reduction of expression from cellular blastoderm through to gastrulation. *D. virilis *shows more intense staining surrounding the midgut during late embryogenesis; however, our *in situ* hybridization protocol resulted in more intense staining of this species generally. So, *CG17734* has conserved maternal expression and ubiquitous expression in late embryogenesis, but there is some variation in the intensity of expression during cellular blastoderm and gastrulation in *D. melanogaster* and *D. simulans*.

*CG11825* is maternally expressed and has a low level of expression during cellular blastoderm stages in *D. melanogaster*, *D. simulans*, and *D. yakuba* (Figure 4). *CG11825* is also weakly expressed throughout later embryogenesis in *D. melanogaster* and *D. simulans*, with more intense staining around the midgut and hindgut; however, it is not expressed here in *D. yakuba*. The modENCODE developmental transcriptome reports that *CG11825* transcript is highest during the early stages of embryogenesis in *D. melanogaster*, but is also expressed at a moderate level throughout embryogenesis, with a second peak at age of 10–12 hours, corresponding to embryonic stages 13–15. The BDGP found that, in *D. melanogaster*, *CG11825 * is expressed maternally and is not expressed in the embryo after the cellular blastoderm stage; however, because BDGP used a probe based on the cDNA of the transcript CG11825-RA, they may have missed the other transcripts that our probe would detect. So, although expression of *CG11825* in early embryogenesis is conserved across the three species, expression in later development is only present in *D. melanogaster* and *D. simulans*, suggesting that *CG11825* has diversified in expression pattern.

Both *D. melanogaster* and *D. simulans*, compared to the other species investigated, showed expression differences in *CG17734* and *CG11825*. These expression differences in *CG17734* and *CG11825* appear to be complementary in these two species: *CG17734* has lower expression during cellular blastoderm and gastrulation, while *CG11825* has expression from cellular blastoderm into later embryogenesis. Transcript levels of *CG17734* and *CG11825* in *D. melanogaster*, determined by modENCODE [57] (Supplemental File 1), also show complementary levels of expression over the course of embryonic development. These observations suggest subfunctionalization of expression pattern [8], which occurs when expression of one gene copy compensates for loss of expression of its paralog. However, it is difficult to make this conclusion. Transcript levels may not accurately reflect posttranscriptional gene activity, and paralogous proteins may not be functionally equivalent.

#### 3.4.2. Parent Gene *Sep2* and Retrogene *Sep5*

**Figure 5:**
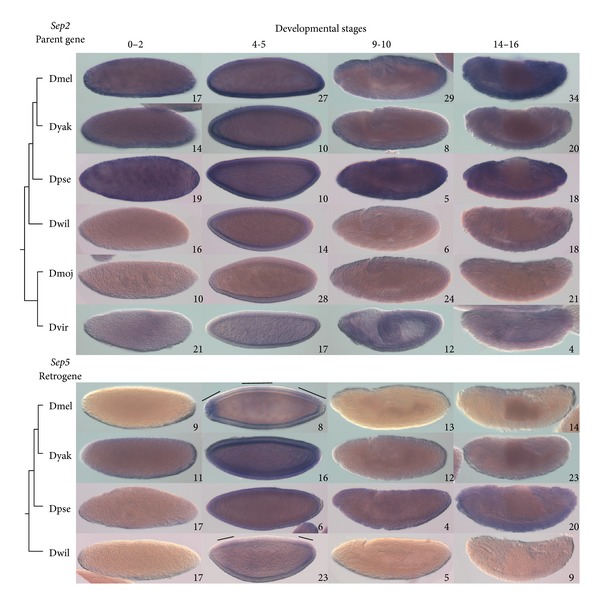
Expression patterns of *Sep2* and *Sep5. *Expression of *Sep2* and *Sep5* was detected in *Drosophila* species by *in situ* hybridization as shown in the top and bottom panels, respectively. Purple coloration indicates presence of mRNA. Cladograms show the relationships of the species investigated. Numbers on individual panels indicate the number of embryos assessed with consistent staining patterns. *Sep5* showed domains of more intense staining during cellular blastoderm stages in *D. melanogaster* and *D. willistoni*; these domains are indicated by black bars. Stage ranges are according to [24]: 0–2, syncytial blastoderm; 3-4, cellular blastoderm; 9-10, during germ-band elongation; 14–16, final embryonic stages. Staining during stages 0–2 indicates presence of maternal transcripts [25]. Dmel, *D. melanogaster*; Dyak, *D. yakuba*; Dpse, *D. pseudoobscura*; Dwil, *D. willistoni*; Dmoj, *D. mojavensis*; Dvir, *D. virilis*.

*Sep2 *transcript is expressed ubiquitously throughout embryonic development in the six species examined (Figure 5). In *D. melanogaster* and *D. yakuba*, there was particularly dark staining in bands of somatic muscle during the final stages of embryonic development. The BDGP found that *Sep2* is expressed ubiquitously throughout embryogenesis in *D. melanogaster*, with more intense staining in the nervous system and dorsal vessel of late-stage embryos which we did not observe. The modENCODE developmental transcriptome database reports *Sep2* expression throughout embryogenesis in *D. melanogaster*.

*Sep5* is expressed during cellular blastoderm in all species examined (Figure 5). In *D. melanogaster*, *Sep5* shows three relatively dark staining bands at the anterior, medial, and posterior regions of the cellular blastoderm. The BDGP found a similar pattern during cellular blastoderm and also found staining along the ventral side of the embryo at the onset of gastrulation and in the endoderm and head mesoderm during gastrulation which we did not observe. In *D. willistoni*, *Sep5* shows darker staining at the anterior and posterior ends during cellular blastoderm. In *D. yakuba* and *D. pseudoobscura*, *Sep5* shows uniform staining during the cellular blastoderm stage and is also ubiquitously expressed throughout embryogenesis. These observations suggest that *Sep5* has diversified in expression pattern, while *Sep2* retained the ancestral expression pattern.

*Sep2* forms a complex with the *Drosophila* group 2B septins, *Sep1* and *pnut*, during embryogenesis in a 1 : 1 : 1 ratio, and these three proteins form filaments *in vitro* [61]. One explanation for the conservation of *Sep2* expression pattern in *Drosophila* species embryos is that all three proteins are required for a functional complex. The BDGP determined that *Sep1* is ubiquitously expressed until the final stages of embryogenesis, and the modENCODE developmental transcriptome shows that all three genes are similarly expressed during embryogenesis. *Sep5* may integrate into the *Sep1*-*pnut*-*Sep2* complex during cellularization too [19]. Protein interaction data show that both *Sep2* and *Sep5* can interact with each other and with *Sep1* and *pnut*, whereas other protein interactions are not shared [62]. Expression of the retrogene *Sep5* during embryogenesis likely has functional consequences for the septin complex and may regulate how the complex interacts with other proteins or membranes.

### 3.5. Sequence Evolution of Three Gene Pairs

TMHMM and Multicoil2 were used to predict additional protein features of the paralogous coding sequences of each gene pair (detailed in Supplemental File 1, visually summarized in Supplemental Figures  2, 3, and  4).* CG8331* and *CG4960* encode proteins with two transmembrane helices, consistent with observations from the yeast homolog Yop1p [14]. The transmembrane helices are well conserved across orthologs and paralogs, except in the case of *CG8331* in *D. ficusphila* which was predicted to have four smaller helices. *CG17734* and *CG11825* appear to encode proteins with two transmembrane helices, consistent with observations from the mouse homolog HIMP1 [16]. The position of the first transmembrane helix of *CG17734* and *CG11825* is absolutely conserved across orthologs and paralogs. The position of the second helix of *CG17734* and *CG11825* is well conserved, but in more distantly related orthologs of *CG17734*, and nearly all orthologs of *CG11825*, this second transmembrane domain was not scored as significant by TMHMM. Both *Sep2* and *Sep5* contain a coiled-coil domain toward the C-terminus; the position of this domain is conserved across orthologs; however, the coiled-coil domain of *Sep5* is predicted not to extend as far as that of *Sep2*. 

We explored the coding sequence evolution of the three gene pairs using various methods (summarized in Table 1, detailed in Supplemental File 1, and visualized using JalView [63] and manually annotated in Supplemental Figures  2, 3, and  4). The branch-site test for positive selection [47] of the PAML package [48] detected positive selection in the parental copies of *Sep2*. In this case the estimated value of *ω* was high, and two amino acid sites were identified by the Bayes Empirical Bayes [49] approach implemented in PAML. Sequence analysis by fixed effects likelihood method (FEL) [52] reveals that the majority of sites in each gene pair have evolved under negative selection. Analysis by mixed effects model of evolution (MEME) [53] detected a few sites under episodic diversifying selection in two of the gene pairs; however, only one instance of episodic diversifying selection, in *Sep2* and *Sep5* near the C-terminus, corresponds to an amino acid difference between the parent and retrogene. So, it appears that the majority of amino acid sites in all three gene pairs evolved under negative selection, indicating constraint on gene function, while positive selection does not appear to be a major factor influencing functional diversification in any of the three gene pairs presented. The multiple sequence alignments of the gene pairs reveal several amino acid sites that are conserved among orthologs, but differ between paralogs. *Sep2* and *Sep5*, which are both classified as group 1B septins [64], show many of these amino acid sites, including differences in the G1 and G3 GTPase domains, the Sep1 motif, and within the coiled-coil domains (Supplemental Figure  4). These likely represent nonsynonymous substitutions that occurred early during the evolution of the three retrogenes, which were then maintained by negative selection.

## 4. Conclusion

Here we have determined embryonic expression patterns and performed sequence analyses to reveal some of the complexities in three cases of retroduplication. Our approach of comparing multiple species with and without a particular retrogene allowed us to investigate how the expression patterns of the gene pairs evolved in relation to one another and whether orthologs evolve similarly across species, unlike studies that only consider differences in transcript level across tissues within a single species [4, 5, 65, 66]. Our results are consistent with the concept that, because retroduplication separates the coding sequence of a parent gene from its transcriptional regulatory elements, retrogenes undergo diversification in expression pattern more readily compared to their parents [3, 66]. The expression and sequence evolution of *Sep2* and *Sep5* seem to reflect Ohno's idea that one gene copy would maintain the ancestral function, allowing the other copy to diverge in function [67]. However, parent gene expression patterns do not have to remain static through evolution, as shown for the *CG17734* and *CG11825* gene pair.

## Supplementary Material

Supplementary File 1 contains a list of alternative transcripts for *CG8331, CG4960, CG17734, CG11825, Sep2,* and *Sep5* in *Drosophila melanogaster*; pairwise sequence alignments; presence or absence of each gene within the 20 Drosophila genome sequences; a description of all orthologs of the six genes within the 20 Drosophila species; primer sequences used to detect transcription using qRT-PCR; primer sequences used to make probes for in situ hybridization; data from the modENCODE developmental transcriptome of *D. melanogaster*; expression data from FlyAtlas; PAML FEL, and MEME results.
Supplementary Figures 2, 3, and 4 contain annotated multiple sequence alignments for the three gene pairs.
Supplementary Figure 5 contains graphs of the expression levels of the three gene pairs as detected by qRT-PCR.
Supplementary File 6 contains the multiple sequence alignments of the three gene pairs in Clustal format.
Supplementary Figure 7 contains examples of background staining for each probe, as determined by using sense probes for *in situ* hybridization.

## Figures and Tables

**Table 1:** Sequence evolution analyses on three gene pairs.

Gene pair	Branch-site test (PAML)			Datamonkey					
	Total no. of sites in codon alignment	Parent as foreground	Retrogene as foreground	Negatively selected sites (FEL)		Sites under episodic diversifying selection (MEME)		Visually identified sites	
		No. of sites^a^	No. of sites^a^	No. of sites^b^	Proportion of total sites	No. of sites^c^	Proportion of total sites	No. of sites^d^	Proportion of total sites
*CG8331* and *CG4960 *	155	N/A	N/A	95	0.613	4	0.026	2	0.013
*CG17734* and *CG11825 *	94	N/A	N/A	67	0.713	0	0	5	0.053
*Sep2* and *Sep5 *	398	2	N/A	356	0.894	3	0.0075	58	0.146

^a^Number of positively selected sites identified by Bayes Empirical Bayes when the null model was rejected; ^b^number of negatively selected sites with *P*  value ≤ 0.05; ^c^number of sites under episodic diversifying selection with *P* value ≤ 0.05; ^d^number of sites with ≥80% amino acid conservation in the parent, ≥80% amino acid conservation in the retrogene, but which were different between parent and retrogene.
